# Phylogenetic analyses of antimicrobial resistant *Corynebacterium striatum* strains isolated from a nosocomial outbreak in a tertiary hospital in China

**DOI:** 10.1007/s10482-023-01855-8

**Published:** 2023-06-27

**Authors:** Yuchuan Li, Jianrong Rong, Chunyan Gao

**Affiliations:** 1 Shanxi Bethune Hospital, Shanxi Academy of Medical Sciences, Tongji Shanxi Hospital, Third Hospital of Shanxi Medical University, Taiyuan, 030032 China; 2 Tongji Hospital, Tongji Medical College, Huazhong University of Science and Technology, Wuhan, 430030 China

**Keywords:** *Corynebacterium striatum*, Antibiotic resistance, Biofilms, 16S rRNA, Phylogenetic analysis

## Abstract

*Corynebacterium striatum* is an emerging, multidrug-resistant pathogen that frequently causes nosocomial infections worldwide. This study aimed to investigate phylogenetic relationship and presence of genes responsible for antimicrobial resistance among *C*.* striatum* strains associated with an outbreak at the Shanxi Bethune Hospital, China, in 2021. Fecal samples were collected from 65 patients with *C. striatum* infection at Shanxi Bethune Hospital between February 12, 2021 and April 12, 2021. *C. striatum* isolates were identified by 16S rRNA and *rpoB* gene sequencing. E-test strips were used to examine the antimicrobial susceptibility of the isolates. Whole-genome sequencing and bioinformatics analysis were employed to assess the genomic features and identify antimicrobial resistance genes of the isolates. Crystal violet staining was conducted to determine the ability of biofilm formation of each isolate. A total of 64 *C. striatum* isolates were identified and categorized into 4 clades based on single nucleotide polymorphisms. All isolates were resistant to penicillin, meropenem, ceftriaxone, and ciprofloxacin but susceptible to vancomycin and linezolid. Most isolates were also resistant to tetracycline, clindamycin, and erythromycin, with susceptibility rates of 10.77, 4.62, and 7.69%, respectively. Genomic analysis revealed 14 antimicrobial resistance genes in the isolates, including *tetW, ermX, and sul1.* Crystal violet staining showed that all isolates formed biofilms on the abiotic surface. Four clades of multidrug-resistant *C. striatum* spread in our hospitals possibly due to the acquisition of antimicrobial resistance genes.

**Supplementary Information:**

The online version contains supplementary material available at 10.1007/s10482-023-01855-8.

## Introduction

*Corynebacterium striatum* is a Gram-positive, normal constituent of the microbiota of human skin and nasal mucosa, and thus has been considered a contaminant of clinical specimens(Lee et al. 2005; Bernard et al. 2013). However, a larger number of invasive infections by *C. striatum* have emerged worldwide in the last five decades (Campanile et al. 2009; Martins et al. 2009; Wong et al. 2010). According to a recent review, a total of 254 cases of *C. striatum* human infections and nosocomial outbreaks have occurred since 1976 (Silva-Santana et al. 2021), affecting both immunosuppressive and immunocompetent individuals(Verma and Kravitz 2016; Lee et al. 2018; Ramos et al. 2019). Nosocomial outbreaks and antibiotic treatment failure of *C. striatum* infection are associated with the resistance of *C. striatum* to multiple antimicrobial agents(Renom et al. 2007). Earlier studies have shown that *C. striatum* isolates were susceptible to many antimicrobial drugs, including tetracycline, β-lactams, and fluoroquinolones(Soriano et al. 1995; Martinez-Martinez et al. 1997; Lagrou et al. 1998). However, more recent reports have indicated increasing multidrug resistance of *C. striatum* strains(Verroken et al. 2014; Yoo et al. 2015; Werth et al. 2016), resulting a mortality rate of nearly 20% in patients with *C. striatum* invasive infection as well as recurrence, superinfection, and limb amputation in many survived patients (Milosavljevic et al. 2021). Thus, it is critically important to unveil the multidrug-resistance mechanisms of *C. striatum*.

Genomic determinants are associated with antimicrobial resistance of *C. striatum.* Horizontal gene transfer plays an essential role in the dissemination of antimicrobial resistance genes within *C. striatum* associated with mobile genomic elements such as plasmids, transposons and insertion sequences(Leyton et al. 2021). For example, *ermX* transported by transposon Tn5432 is associated with the resistance of *C. striatum* to macrolides, lincosamides, and streptogramin(Tauch et al. 2000). Plasmid pJA144188 and insertion sequences IS3502, IS3503, and IS3504 can transport *tetW* that confers tetracycline resistance(Schroder et al. 2012; Nudel et al. 2018; Wang et al. 2019). Whole-genome sequencing compared with those available in the GenBank database is more accurate method for the identification of *C. striatum* compared with MALDI-TOF, Vitek 2 and API (Milosavljevic et al. 2021). When combined with bioinformatics analysis, whole-genome sequencing serves as a powerful tool to understand the genomic features and track nosocomial transmission of pathogens(Quainoo et al. 2017). Genomic analysis has revealed that the acquisition of antimicrobial resistance genes through genetic transfer contributes to *C. striatum* hospital infections(Alibi et al. 2017; Wang et al. 2019). In addition, *C. striatum* strains can form biofilms on abiotic substrates, including indwelling medical devices and steel surfaces, thus facilitating the tolerance of *C. striatum* to disinfectants and antiseptics agents(Souza et al. 2015; Souza et al. 2020). Therefore, identification of antimicrobial resistance genes and examination of the biofilm-forming ability of *C. striatum* strains may help elucidate the multidrug-resistance mechanisms of *C. striatum.*

In this study, we investigated the genomic diversity and the multidrug resistance mechanism of *C. striatum* that caused a nosocomial outbreak at Shanxi Bethune Hospital, China, in 2021. Our results may provide valuable information for the prevention and management of *C. striatum* infection in the hospital.

## Materials and methods

### Study design and ethical approval

This study was approved by the local medical ethical committee (YXLL-KY-2021–015; Shanxi, China). All experiments were performed following relevant regulations. Fecal samples were obtained from 65 patients infected with *C. striatum.* Patients were admitted to Shanxi Bethune Hospital from February 12, 2021 to April 12, 2021. The inclusion criteria were: (1) patients stayed in the hospital at least 3 days; (2) patients showed clinical symptoms of respiratory or urinary tract infection or had pain, redness, or swelling around the insertion site of venous catheter; (3) the sputum or bronchial aspirate specimen contained ≥ 25 white blood cells/high-power filed and the number of *C. striatum* clones isolated from sputum ≥ 10^6^ CFU/ml in quantitative sputum culture. (4) the urine sample contained > 5 white blood cells/ high-power filed and the number of *C. striatum* clones isolated from urine ≥ 10^4^ CFU/ml in quantitative urine culture (5) the number of *C. striatum* clones isolated from venous catheter ≥ 15 CFU/5 cm.

## Isolation and identification of *C. striatum*

Clinical specimens were inoculated on agar plates (Autobio Diagnostics, Zhengzhou, Henan, China) and cultured at 35 °C for 24–72 h in an atmosphere of 5% CO_2_. Genomic DNA was isolated from the colonies using a Gentra Puregene Yeast/Bact Kit (Qiaqen, Hilden, Germany) according to the manufacturer’s instruction. The quality of genomic DNA was assessed by electrophoresis on 1% agarose gels and OD 260/280 ratio. The DNA concentration was measured using a Qubit® DNA assay kit (Invitrogen, Waltham, MA, USA). All isolates were initially identified as *C. striatum* by MALDI-TOF using the Microflex LT/SH MS system (Bruker Daltonics, Bremen, Germany) in IVD HCCA solution, followed by 16 s rRNA sequencing (Novogene, Beijing, China) and PCR amplification of *rpoB* encoding RNA polymerase β subunit. The 16 s rRNA sequencing results were aligned to the complete genomic sequence of *C. striatum* in NCBI (https://www.ncbi.nlm.nih.gov/) using the BLAST algorithm and the Ribosomal Database Project II (http://rdp.cme.msu.edu/). All the isolates were confirmed as *C. striatum* with similarity ≥ 99%. PCR amplification of *rpoB* was conducted on a Mastercycler® nexus thermal cycler (Eppendorf, Hamburg, Germany) using primers 5´-CGTATGAACATYGGBCAGGT-3ʹ (forward) and 5ʹ -TCCATYTCRCCRAARCGCTG-3ʹ (reverse) (Sangon Biotech, Shanghai, China). The thermal cycles were 94 °C for 5 min, 30 cycles of 90 °C for 30 s, 64 °C for 30 s, and 72 °C for 1 min, followed by 72 °C for 2 min. The *rpoB* sequence of each isolate was aligned to the *rpoB* sequence of *C. striatum* in the GenBank database. All the isolates were confirmed as *C. striatum* with similarities ≥ 99%.

## Whole-genome sequencing

A total of 0.2 μg DNA per sample was used to prepare the DNA library. The sequencing library was generated using NEB Next® Ultra™ DNA library prep kit for Illumina (New England Biolabs, Ipswich, MA, USA) following the manufacturer’s instructions. The index codes were added to each sample. Briefly, genomic DNA was sonicated into fragments of 350 bp. Then, DNA fragments were end-polished, A-tailed, and ligated with the full-length adapter for Illumina sequencing, followed by PCR amplification. The PCR products were purified by the AMPure XP system (Beckman Coulter, Beverly, USA), and the DNA concentration was measured using a Qubit®3.0 flurometer (Invitrogen). The DNA library was analyzed for size distribution using Agilent 2100 Bioanalyzer (Agilent, Santa Clara, CA, USA) and quantified by real-time PCR (> 2 nM). The clustering of the index-coded samples was performed on a cBot cluster generation system using Illumina PE cluster kit (Illumina, USA) according to the manufacturer’s instructions. Then, the DNA library was sequenced on the Illumina platform to generate 150 bp paired-end reads.

## Phylogenetic analysis

A single nucleotide polymorphism (SNP)-based phylogenetic analysis was performed using the whole genome sequence of *C. striatum* RefCP021252 as the reference. The Trimmomatic software^31^ was used to filter low-quality reads before SNP extraction. The clean reads of the isolates were aligned to the reference genome using SPAdes software (http://bioinf.spbau.ru/en/spades) under the default parameters. The Snippy pipeline, version 3.0 (https://github.com/tseemann/snippy), was used for read mapping and variant calling. SNPs were identified to reveal the phylogenomic relationship of *C. striatum* isolates using CSI Phylogeny as previously described(Kaas et al. 2014; Ahrenfeldt et al. 2017). The Z-score cutoff value was 1.96. SNPs within 10 base pairs were removed. A maximum likelihood tree was generated by MEGA Software (Kumar et al. 1994). A phylogenetic test was conducted using the assembled contigs from the root strain as reference genome, SNP pruning disabled(Efron et al. 1996).

## Identification of antimicrobial resistance genes

Clean reads from whole-genome sequencing were obtained by removing the adapters, the reads with unknown bases > 10%, and the reads with low-quality bases (Q-value < 5) > 50% using the Trimmomatic Software(Bolger et al. 2014), followed by assembly using SPAdes(Bankevich et al. 2012). Antimicrobial resistance genes were identified using the Center for Genomic Epidemiology server (http://www.genomicepidemiology.org/) (Hendriksen et al. 2019). An antimicrobial resistance gene was considered present in the isolate when the sequence exhibited ≥ 99.9% identity to that in the GeneBank (www.ncbi.nlm.nih.gov/genbank/). A heatmap of the antimicrobial resistance genes was generated using the R pheatmap package.

## Antimicrobial susceptibility test

Antimicrobial susceptibility was determined using E-test strips in Mueller–Hinton agar plates (Autobio Diagnostics, Zhengzhou, Henan, China) as previously described(Ramos et al. 2019). E-test strips containing penicillin, meropenem, ceftriaxone, tetracycline, ciprofloxacin, clindamycin, erythromycin, gentamicin, and vancomycin were from Autobio Diagnostics. E-test strips containing rifampicin and linezolid were from Beijing Weitaike Biotechnology (Beijing, China). The plates were incubated at 37 °C in an atmosphere of 5% CO_2_ for 24 h. The minimum inhibitory concentration (MIC) was determined and interpreted according to The Clinical and Laboratory Standards Institute (CLSI) M45 guidelines (JA Hindler 2016).

## Semiquantitative analysis of biofilm formation

Ten microliter of 0.5 McFarland bacterial suspension was plated in 200 uL trypticase soy broth (Sangon Biotech) in a 96-well plate and cultured for 24 h at 37°C. After removing the broth, the adherent bacteria (biofilms) were washed with ice-cold phosphate-buffered saline, fixed with 200 μL methanol for 15 min, and stained with 200 μL 0.1% crystal violet (Sangon Biotech) for 5 min. The absorbance (OD value) was determined at 595 nm. The experiment was performed in quadruplets. Trypticase soy broth was used as a negative control.

## Statistical analysis

Categorical data were expressed as percentages. Data were analyzed using SPSS 26 (Armonk, NY, USA). Quantitative data were expressed as the mean ± standard deviation. The differences between the two groups were compared using one-way analysis of variance and Chi-square test. A *P* value < 0.05 was considered statistically significant.

## Results

### Identification of *C. striatum* isolates

A total of 64 *C. striatum* isolates were obtained from 65 patients infected with *C. striatum*, including 18 (27.69%) females and 47 (72.31%) males predominantly aged between 51 and 79 years (75.38%). The clinical features of the patients were summarized in Table 1. The presence of *C. striatum* was mainly confirmed in sputum specimens (51/78.46%), followed by bronchial aspirate (9/13.84%), venous catheter (2/3.08%), wound secretion (2/3.08%), and urine (1/1.54%) specimens. The isolates were from 9 wards, with neurosurgery wards (30/47.68%), intensive care units (9/13.85%), and general medicine wards (8/12.31%) representing the most common locations. The most common preexisting conditions of the patients were cerebral hemorrhage (44/67.69%), hypertension (32/49.23%), and pulmonary inflammation (32/49.23%). All patients received at least one type of antibiotic treatment. Of these, 23 (35.38%) received two types of antibiotics, and 27 (41.54%) received three or more types of antibiotics. In addition, ward distribution and strain acquisition time of *C. striatum* infection were shown in supplementary table 1. These results suggest that males, patients with critical underlying conditions, patients exposed to multiple antibiotics and patients over 50 years old were higher proportion compared with counterparts.

**Table 1:** Demographic and clinical features of patients (n = 65)

Parameter	Number (%)
*Age(years)*	
≤ 50	9 (13.85)
51–79	49 (75.38)
≥ 80	7 (10.77)
*Gender*	
Female	18 (27.69)
Male	47 (72.31)
*Hospital ward(s)*	
Neurosurgery	30 (46.15)
Intensive care units (ICU)	9 (13.85)
General medicine	8 (12.31)
Department of Respiratory and Critical Care Medicine(PCCM)	6 (9.23)
Rehabilitation medicine	5 (7.69)
Lymphatic tumour	3 (4.62)
Neurology	2 (3.08)
Liver surgery	1 (1.54)
Digestive internal medicine	1 (1.54)
*Specimens*	
Sputum	51 (78.46)
Endotracheal aspirate	9 (13.84)
Venous catheter	2 (3.08)
Wound secretion	2 (3.08)
Urine	1 (1.54)
*Intravenous antibiotics*	
Three or more kinds of antibiotics	27 (41.54)
Two kinds of antibiotics	23 (35.38)
Only an antibiotic	15 (23.08)
*Underlying diseases*	
Cerebral hemorrhage	44 (67.69)
Hypertension	32 (49.23)
Pulmonary inflammation	32 (49.23)
Disturbance of consciousness	12 (18.46)
Diabetes	11 (16.92)
Cancer	10 (15.38)
Respiratory failure	7 (10.77)
Cardiac insufficiency	6 (9.23)
Compound fractures	3 (4.62)
Urinary tract infection	2 (3.08)
Gastrointestinal hemorrhage	1 (1.54)

## Phylogenetic analysis

**Fig. 1:**
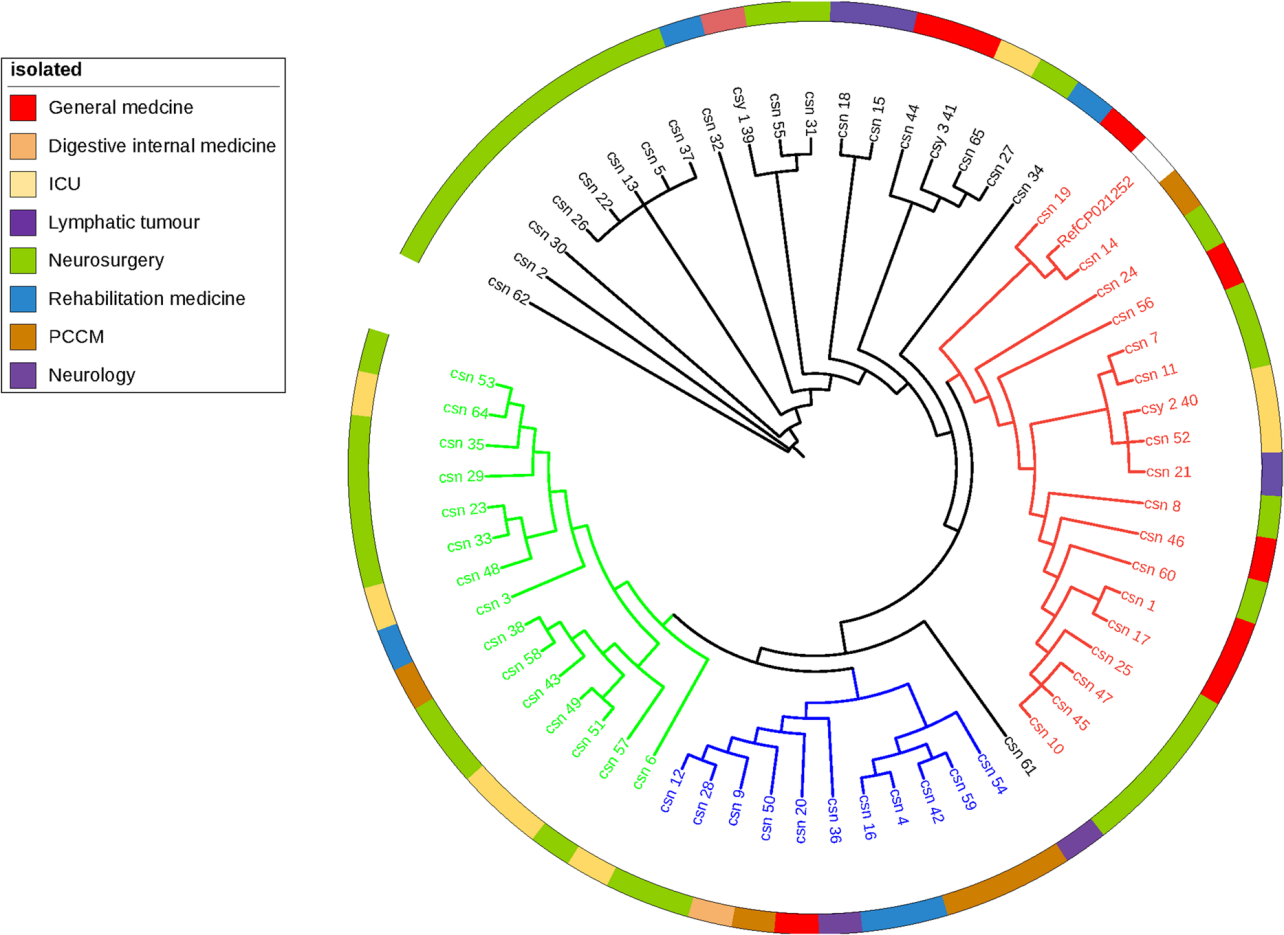
Phylogenomic analysis of *C. striatum* isolates. Phylogenomic analysis was performed using CSI Phylogeny. The assembled contigs from the root strain was uesd as reference genome, SNP pruning disabled. *C. striatum* CP021252 was used as the reference sequence. Clades I (n = 20), II (n = 18), III (n = 11), and IV (n = 15) are indicated in black, red, blue, and green, respectively. csy: *C. striatum* obtained from steriles amples; csn: *C. striatum* obtained from non-sterile samples. (Color figure online)

To characterize the genomic diversity of the *C. striatum* isolates, we performed an SNP-based phylogenetic analysis. As shown in Fig. 1, the isolates were clustered into four clades compared with the referenc genome (RefCP021252), with clade I containing the highest number of isolates (n = 20), followed by clade II, IV, and III containg 18, 15, and 11 isolates, respectively. Of the 64 isolates, isolate 62 showed the greatest evolution. Clade III exhibited the greatest diversity compared with other clades. Furthermore, clades I and III circulted in 6 wards, whereas clades II and IV circulated in 4 wards. The neurosurgery ward was the only ward that contained all the 4 clades and was the main ward in clade I (Supplementary table2). In addition, we conducted the correlation analysis between gene clades and the neurosurgery ward. Nevertheless, the result was negative (Supplementary table3). A long-term multicenter investigation is urgent in the future.

## Antimicrobial susceptibility testing

To assess the antibiotic resistance of the isolates, we determined the antimicrobial susceptibility. All isolates were resistant to penicillin, meropenem, ceftriaxone, and ciprofloxacin but susceptible to vancomycin and linezolid. Most isolates were also resistant to tetracycline, clindamycin, and erythromycin, with susceptibility rates of 10.77, 4.62, and 7.69%, respectively. However, most isolates were susceptible to gentamicin and rifampicin, with susceptibility rates of 75.38 and 96.92%, respectively. The MICs and susceptibility rates were summarized in Table 2. These results indicate that the *C. striatum* isolates in this study are resistant to multiple antibiotics. Vancomycin and linezolid are the antibiotics of choice for the treatment of *C. striatum* infections.

**Table 2:** Antimicrobial susceptibility test

Antibiotics	Dose range (µg/mL)	MIC (µg/mL)			Percentage of susceptible isolates (%)
		S	I	R	
Penicillin	0.002–32	≤ 0.12	0.25–4	≥ 4	0
Meropenem	0.002–32	≤ 0.25	0.5	≥ 1	0
Ceftriaxone	0.002–32	≤ 1	2	≥ 4	0
Tetracycline	0.016–256	≤ 4	8	≥ 16	10.77
Ciprofloxacin	0.002–32	≤ 1	2	≥ 4	0
Clindamycin	0.016–256	≤ 0.5	1–2	≥ 4	4.62
Erythromycin	0.016–256	≤ 0.5	1	≥ 2	7.69
Gentamicin	0.016–256	≤ 4	8	≥ 16	75.38
Rifampicin	0.008–32	≤ 1	2	≥ 4	96.92
Vancomycin	0.016–256	≤ 2	–	–	100
Linezolid	0.016–256	≤ 2	–	–	100

MIC, minimum inhibitory concentration; S, susceptible; I, intermediate; R, resistant

## Identification of antimicrobial resistance genes

**Fig. 2:**
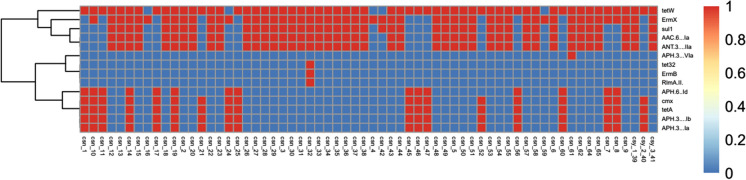
Heatmap of antimicrobial resistance genes in *C. striatum* isolates. Antimicrobial resistance genes were identified using the Center for Genomic Epidemiology server (http://www.genomicepidemiology.org) based on whole-genome analysis. A heatmap was generated to illustrate antimicrobial resistance genes in each isolate. Red (1) indicates the presence of antimicrobial resistance genes; blue (0) indicates the absence of antimicrobial resistance genes. The vertical axis represents antimicrobial resistance genes. The horizontal axis represents *C. striatum* isolates. csy: *C. striatum* obtained from sterile samples; csn: *C. striatum* obtained from non-sterile samples. (Color figure online)

To investigate the underlying mechanism of antibiotic resistance of the isolates, we sought to identify antimicrobial resistance genes. Genomic analysis revealed 14 antimicrobial resistance genes in the isolates (Fig. 2). Of these genes, *APH(3ʹ)-*Via was only present in csn-61 isolate. *ErmB*, *tet32* and *RlmAII* were only present in csn-32 isolate. *TetW* was present in 59 isolates, followed by *ermX* in 52 isolates, *sul1* in 45 isolates. *TetW* conferring resistance to tetracycline was the most frequent (Hahn et al. 2016), followed by *ermX* conferring resistance to erythromycin and clindamycin(Campanile et al. 2009), *sul1* conferring resistance to sulfamethoxazole (Yao et al. 2020), *aac(6*ʹ*)-Ia*, *ant (3″)-IIa*, *aph(3')-Ia*, and *aph(3')-Ib* conferring resistance to aminoglycosides(Miro et al. 2013; Nie et al. 2014), *tetA* conferring resistance to tetracycline(Nguyen et al. 1983), *cmx* conferring resistance to chloramphenicol and lincomycin(Peterson and Kaur 2018), and *aph(6)-Id* conferring resistance to streptomycin(Ashenafi et al. 2014). Besides, we observed the presence of *aph(3)-*VIa, *tet32*, and *ErmB* conferring resistance to streptomycin, tetracycline, and erythromycin in isolate 32 as well as *RImA*^*II*^ conferring resistance to tylosin(Liu and Douthwaite 2002)in isolate 61. Overall, 61 (93.8%) of the isolates contained more than five antibiotic-resistance genes (Supplementary table2). These results suggest that the *C. striatum* isolates evolve to multidrug-resistant clones by acquiring antimicrobial resistance genes. We also discovered csn-14 and csn-19 isolates in clades II contained ten identical antimicrobial resistance genes; isolates csn-4, csn-16 and csn-59 contained the same *ermX* in clade III (Supplementary table2). The correlation analysis between gene clades and numbers of antimicrobial resistance genes was conducted (*P* < 0.05). It was potential of different patterns that these genes distributed among four different clades (Supplementary table3). Isolates csn-7, csn-8 and csn-10 from neurosurgery ward contained seven identical antimicrobial resistance genes. This discovery implicated small clonal circulations in our hospital.

## Semiquantitative analysis of biofilm formation

**Fig. 3:**
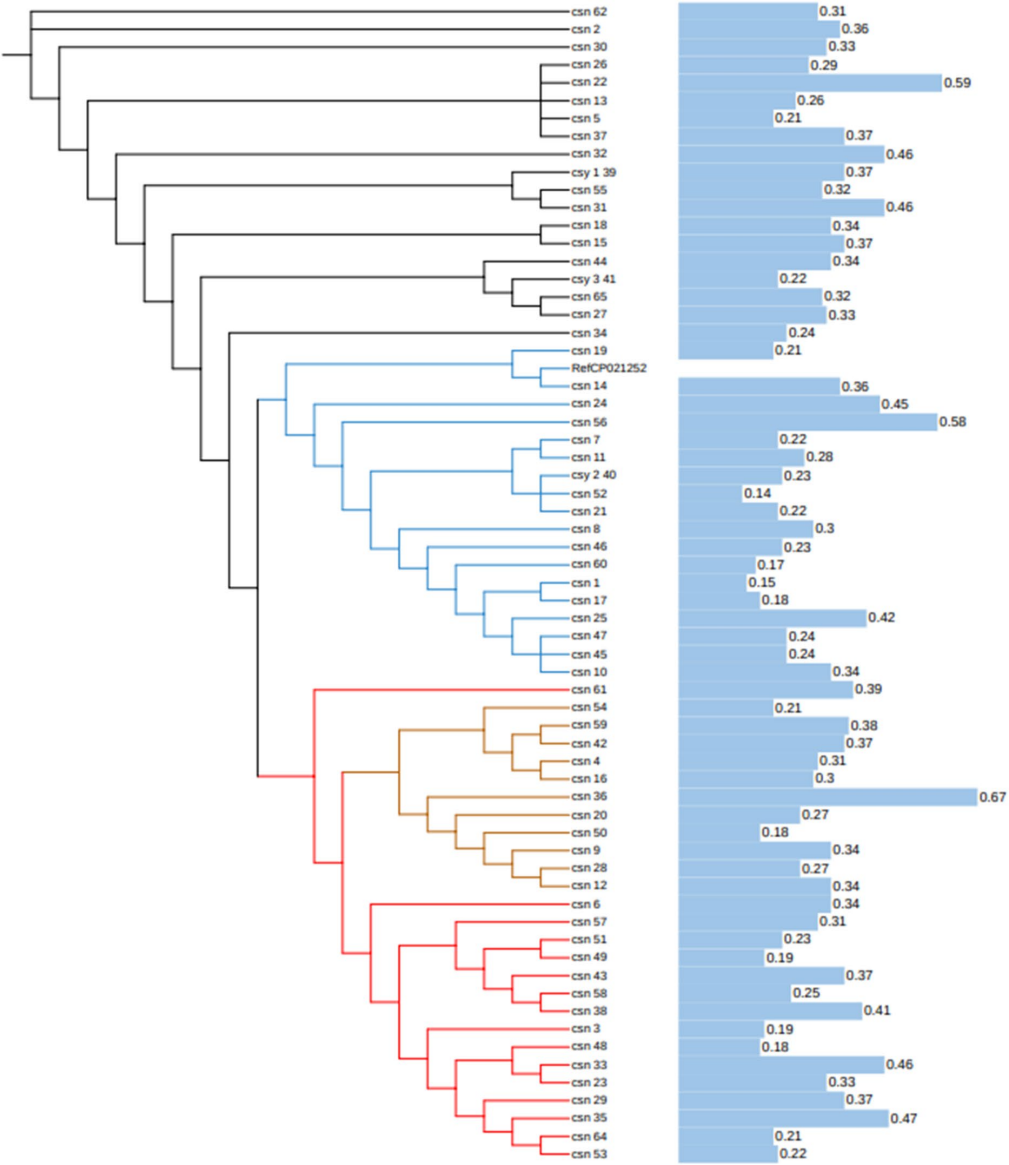
Semiquantitative analysis of biofilm formation. Ten microliters of 0.5 McFarland *C. striatum* suspension was incubated in 200 uL trypticase soy broth in a 96-well plate for 24 h at 37 °C. Crystal violet staining was performed, and the absorbance (OD value) was measured at 595 nm to determine biofilm formation. Trypticase soy broth was used as a negative control. Black, blue, brown, and red branches indicate four clades. The length of the blue horizontal column indicates the OD value shown alongside. (Color figure online)

Considering the critical contribution of bacterial biofilms to antibiotic resistance(Sharma et al. 2019), we determined the biofilm formation of the isolates. Crystal violet staining showed that all isolates formed biofilms with OD values ranging from 0.14 to 0.67 (Fig. 3). We divided all strains into two groups according to OD value of biofilms (< 0.35 or ≥ 0.35) and explored if gene clades was associated with biofilm-forming ability of the isolates. As a pity, the result was negative (Supplementary table3). This requires the advanced method of biofilm detection and expansion the size of the study.

## Discussion

In this study, we obtained 64 *C. striatum* isolates from 65 patients with *C. striatum* infection in a tertiary hospital in China. Sample 63 was excluded due to inadequate amounts of DNA (< 200 ng/uL) for subsequent analysis. SNP-based phylogenetic analysis categorized the isolates into 4 clades. Antimicrobial susceptibility test revealed that the isolates were resistant to multiple antibiotics due to the presence of antimicrobial resistance genes. The distribution pattern of antibiotic-resistance genes was different among 4 clades. Three isolates shared the same genetic background in neurosurgery and two isolates with ten identical antimicrobial resistance genes belonged to clade II. A circulation of single-clone probably existed in our hospital. Therefore, a comprehensive infection control measure is urgent in the future.

*C. striatum* isolates have been frequently reported in sputum and bronchial aspirate(Hahn et al. 2016; Wang et al. 2019). Consistently, in this study, the presence of *C. striatum* was mainly confirmed in sputum specimens (51/78.46%) and bronchial aspirate (9/13.84%). Recently, bloodstream and catheter-related *C. striatum* infections have attracted attention due to the increased number of cases(Ramos et al. 2019; Souza et al. 2019). Indeed, we also obtained *C. striatum* isolates from the venous catheter (2/3.08%), possibly resulting from the biofilm-forming ability of the isolates on abiotic surfaces(Souza et al. 2019). Studies have demonstrated that most *C. striatum* infections occur in immunocompromised patients and patients with underlying medical problems or skin damage(Superti et al. 2009; Diez-Aguilar et al. 2013). Invasive medical devices, long-term exposure to broad-spectrum antibiotics, and long-term hospitalization are also risk factors for *C. striatum* infections(Lee et al. 2005). Similarly, our results showed that males (72.3%), elderly patients over 50 years old (86.2%), patients in the neurosurgery ward, patients with cerebral hemorrhage (67.7%), and those receiving at least two antibiotics (76.9%) were more likely to develop *C. striatum* infection compared with their counterparts (Supplementary table4). Thus, a standard surveillance program for these patients is recommended for the prevention and early diagnosis of *C. striatum* infections.

Studies have shown that *C. striatum* isolates are resistant to penicillin, meropenem, ceftriaxone, tetracycline, clindamycin, erythromycin, and ciprofloxacin(Hahn et al. 2016; McMullen et al. 2017). Consistently, all isolates in this study were resistant to penicillin, meropenem, ceftriaxone, and ciprofloxacin. Most isolates were also resistant to tetracycline, clindamycin, and erythromycin, with susceptibility rates of 10.77%, 4.62%, and 7.69%, respectively. However, despite the resistance to multiple antibiotics, *C. striatum* isolates in this study were susceptible to vancomycin, linezolid, gentamicin, and rifampicin, with susceptibility rates of 100%, 100%, 75.38%, and 96.92%, respectively, consistent with previous findings(Navas et al. 2016; Alibi et al. 2017). Therefore, vancomycin and linezolid are the antibiotics of choice for the treatment of *C. striatum* infections, sometimes in combination with other antibiotics such as gentamicin (Rufael and Cohn 1994; Marull and Casares 2008).

*C. striatum* may evolve into persistent and dominant clones within the hospital by acquiring antimicrobial resistance genes(Wang et al. 2019; Wang et al. 2021). Our genomic analysis identified 14 antimicrobial resistance genes in the isolates. Of these genes, *tetW, ermX, sul1*, *aac(6*ʹ*)-Ia*, and *ant (3″)-IIa* were expressed in more than 75% of the isolates, suggesting that resistance to antimicrobials may be acquired by horizontal gene transfer within the hospital. In addition, 61 (93.8%) isolates carried at least 5 antimicrobial resistance genes, explaining the resistance of the isolates to multiple antibiotics.

The ability of biofilm formation allows *C. striatum* to adhere to living and artificial surfaces to resist antibiotics and host immune factors, playing an important role in the virulence potential of *C. striatum*(Souza et al. 2019). Multidrug-resistant *C. striatum* strains exhibit greater abilities to adhere to both hydrophilic and hydrophobic abiotic surfaces than multidrug-sensitive counterparts(Souza et al. 2015), suggesting that biofilm production contribute to multidrug resistance of *C. striatum.* All the 64 *C. striatum* isolates in this study formed biofilms on the abiotic surface, suggesting that *C. striatum* isolates were resistant to multiple antibiotics at least partially due to the biofilm-forming ability of the isolates.

This study has some limitations that will be addressed in the future. First, the presence of antimicrobial resistance genes needs to be verified using molecular methods such as PCR. Second, scanning electron microscopy will be employed to demonstrate the production of biofilms.

## Conclusion

In conclusion, four clades of multidrug-resistant C. striatum spread at our hospital between February and April 2021. Horizontal transfer of antimicrobial resistance genes and the ability of biofilm formation are likely responsible for the transmission of C. striatum in the hospital. These findings may provide helpful information about the prevention and treatment of nosocomial outbreak of C. striatum. The raw sequence data reported in this paper have been deposited in the Genome Sequence Archive (Genomics, Proteomics & Bioinformatics 2021) in National Genomics Data Center (Nucleic Acids Res 2022), China National Center for Bioinformation / Beijing Institute of Genomics, Chinese Academy of Sciences (GSA: CRA008854) that are publicly accessible at https://ngdc.cncb.ac.cn/gsa.

## Supplementary Information

Below is the link to the electronic supplementary material.

Supplementary file1 (DOCX 18 kb)

Supplementary file2 (DOCX 21 kb)

Supplementary file3 (DOCX 16 kb)

Supplementary file4 (DOCX 16 kb)

## Data Availability

The datasets generated during and/or analysed during the current study are available from the corresponding author on reasonable request.
