# Supplementary material for: Phylogenetic analyses of antimicrobial resistant Corynebacterium striatum strains isolated from a nosocomial outbreak in a tertiary hospital in China
Source: Antonie Van Leeuwenhoek. 2023 Jun 27;116(9):907–18. doi: 10.1007/s10482-023-01855-8 (PMC10371919; doi:10.1007/s10482-023-01855-8)
Supplement: Supplementary file 1 — Supplementary file1 (DOCX 18 kb) [file 10482_2023_1855_MOESM1_ESM.docx]

| **Strain ID** | **Ward** | **Sample acquisition time** |
| --- | --- | --- |
| csn-1 | General medcine | 2021.3.5 |
| csn-2 | Neurosurgery | 2021.3.9 |
| csn-3 | Rehabilitation medicine | 2020.3.1 |
| csn-4 | Rehabilitation medicine | 2021.2.25 |
| csn-5 | Neurosurgery | 2021.3.11 |
| csn-6 | ICU | 2021.3.12 |
| csn-7 | Neurosurgery | 2021.3.11 |
| csn-8 | Neurosurgery | 2021.3.10 |
| csn-9 | Digestive internal medicine | 2021.3.12 |
| csn-10 | Neurosurgery | 2021.3.13 |
| csn-11 | Neurosurgery | 2021.3.14 |
| csn-12 | Neurosurgery | 2021.3.14 |
| csn-13 | Neurosurgery | 2021.2.20 |
| csn-14 | PCCM | 2021.3.15 |
| csn-15 | Lymphatic tumour | 2021.3.15 |
| csn-16 | Rehabilitation medicine | 2021.3.15 |
| csn-17 | General medcine | 2021.3.17 |
| csn-18 | Lymphatic tumour | 2021.3.17 |
| csn-19 | General medcine | 2021.3.17 |
| csn-20 | General medcine | 2021.3.17 |
| csn-21 | Lymphatic tumour | 2021.3.17 |
| csn-22 | Neurosurgery | 2021.3.18 |
| csn-23 | Neurosurgery | 2021.3.18 |
| csn-24 | Neurosurgery | 2021.3.18 |
| csn-25 | Neurosurgery | 2021.3.21 |
| csn-26 | Neurosurgery | 2021.3.22 |
| csn-27 | Neurosurgery | 2021.3.22 |
| csn-28 | Neurosurgery | 2021.3.22 |
| csn-29 | Neurosurgery | 2021.3.23 |
| csn-30 | Neurosurgery | 2021.3.23 |
| csn-31 | Neurosurgery | 2021.3.25 |
| csn-32 | Rehabilitation medicine | 2021.3.24 |
| csn-33 | Neurosurgery | 2021.3.24 |
| csn-34 | Rehabilitation medicine | 2021.3.24 |
| csn-35 | Neurosurgery | 2021.3.24 |
| csn-36 | Neurology | 2021.3.24 |
| csn-37 | Neurosurgery | 2021.3.25 |
| csn-38 | PCCM | 2021.3.23 |
| csy1-39 | Liver surgery | 2021.3.13 |
| csy2-40 | ICU | 2021.3.16 |
| csy3-41 | General medcine | 2021.3.18 |
| csn-42 | PCCM | 2021.3.27 |
| csn-43 | Neurosurgery | 2021.3.27 |
| csn-44 | General medcine | 2021.3.28 |
| csn-45 | Neurosurgery | 2021.3.28 |
| csn-46 | General medcine | 2021.4.4 |
| csn-47 | Neurosurgery | 2021.4.5 |
| csn-48 | ICU | 2021.4.5 |
| csn-49 | ICU | 2021.4.5 |
| csn-50 | PCCM | 2021.4.5 |
| csn-51 | ICU | 2021.4.5 |
| csn-52 | ICU | 2021.4.5 |
| csn-53 | Neurosurgery | 2021.4.5 |
| csn-54 | PCCM | 2021.4.5 |
| csn-55 | Neurosurgery | 2021.4.5 |
| csn-56 | General medcine | 2021.4.6 |
| csn-57 | Neurosurgery | 2021.4.7 |
| csn-58 | Neurosurgery | 2021.4.7 |
| csn-59 | PCCM | 2021.4.7 |
| csn-60 | Neurosurgery | 2021.4.9 |
| csn-61 | Neurology | 2021.4.7 |
| csn-62 | Neurosurgery | 2021.4.9 |
| csn-63 | ICU | 2021.4.7 |
| csn-64 | ICU | 2021.4.7 |
| csn-65 | ICU | 2021.4.9 |

Supplementary table 1 Ward distribution and strain acquisition time of *Corynebacterium striatum* infection
