# Supplementary material for: Phylogenetic analyses of antimicrobial resistant Corynebacterium striatum strains isolated from a nosocomial outbreak in a tertiary hospital in China
Source: Antonie Van Leeuwenhoek. 2023 Jun 27;116(9):907–18. doi: 10.1007/s10482-023-01855-8 (PMC10371919; doi:10.1007/s10482-023-01855-8)
Supplement: Supplementary file 2 — Supplementary file2 (DOCX 21 kb) [file 10482_2023_1855_MOESM2_ESM.docx]

| **Gene clades** | **samples** | **hospital unites** | **OD value**  **(biofilm)** | **numbers of resistance gene** |
| --- | --- | --- | --- | --- |
| genotype1 | csn 62 | Neurosurgery | 0.31 | 5 |
| genotype1 | csn 2 | Neurosurgery | 0.36 | 5 |
| genotype1 | csn 30 | Neurosurgery | 0.33 | 5 |
| genotype1 | csn 26 | Neurosurgery | 0.29 | 5 |
| genotype1 | csn 22 | Neurosurgery | 0.59 | 5 |
| genotype1 | csn 13 | Neurosurgery | 0.26 | 5 |
| genotype1 | csn 5 | Neurosurgery | 0.21 | 5 |
| genotype1 | csn 37 | Neurosurgery | 0.37 | 5 |
| genotype1 | csn 32 | Rehabilitation medicine | 0.46 | 8 |
| genotype1 | csy 1 39 | Liver surgery | 0.37 | 5 |
| genotype1 | csn 55 | Neurosurgery | 0.32 | 5 |
| genotype1 | csn 31 | Neurosurgery | 0.46 | 5 |
| genotype1 | csn 18 | Lymphatic tumour | 0.34 | 5 |
| genotype1 | csn 15 | Lymphatic tumour | 0.37 | 5 |
| genotype1 | csn 44 | General medcine | 0.34 | 5 |
| genotype1 | csy 3 41 | General medcine | 0.22 | 5 |
| genotype1 | csn 65 | ICU | 0.32 | 5 |
| genotype1 | csn 27 | Neurosurgery | 0.33 | 5 |
| genotype1 | csn 34 | Rehabilitation medicine | 0.24 | 5 |
| genotype1 | csn 61 | Neurology | 0.39 | 6 |
| genotype2 | csn 19 | General medcine | 0.21 | 10 |
| genotype2 | csn 14 | PCCM | 0.36 | 10 |
| genotype2 | csn 24 | Neurosurgery | 0.45 | 6 |
| genotype2 | csn 56 | General medcine | 0.58 | 6 |
| genotype2 | csn 7 | Neurosurgery | 0.22 | 7 |
| genotype2 | csn 11 | Neurosurgery | 0.28 | 6 |
| genotype2 | csy 2 40 | ICU | 0.23 | 5 |
| genotype2 | csn 52 | ICU | 0.14 | 5 |
| genotype2 | csn 21 | Lymphatic tumour | 0.22 | 5 |
| genotype2 | csn 8 | Neurosurgery | 0.3 | 7 |
| genotype2 | csn 46 | General medcine | 0.23 | 6 |
| genotype2 | csn 60 | Neurosurgery | 0.17 | 6 |
| genotype2 | csn 1 | General medcine | 0.15 | 6 |
| genotype2 | csn 17 | General medcine | 0.18 | 6 |
| genotype2 | csn 25 | Neurosurgery | 0.42 | 6 |
| genotype2 | csn 47 | Neurosurgery | 0.24 | 6 |
| genotype2 | csn 45 | Neurosurgery | 0.24 | 6 |
| genotype2 | csn 10 | Neurosurgery | 0.34 | 7 |
| genotype3 | csn 54 | PCCM | 0.21 | 5 |
| genotype3 | csn 59 | PCCM | 0.38 | 1 |
| genotype3 | csn 42 | PCCM | 0.37 | 2 |
| genotype3 | csn 4 | Rehabilitation medicine | 0.31 | 1 |
| genotype3 | csn 16 | Rehabilitation medicine | 0.3 | 1 |
| genotype3 | csn 36 | Neurology | 0.67 | 5 |
| genotype3 | csn 20 | General medcine | 0.27 | 5 |
| genotype3 | csn 50 | PCCM | 0.18 | 5 |
| genotype3 | csn 9 | Digestive internal medicine | 0.34 | 5 |
| genotype3 | csn 28 | Neurosurgery | 0.27 | 5 |
| genotype3 | csn 12 | Neurosurgery | 0.34 | 5 |
| genotype4 | csn 6 | ICU | 0.34 | 5 |
| genotype4 | csn 57 | Neurosurgery | 0.31 | 5 |
| genotype4 | csn 51 | ICU | 0.23 | 5 |
| genotype4 | csn 49 | ICU | 0.19 | 5 |
| genotype4 | csn 43 | Neurosurgery | 0.37 | 5 |
| genotype4 | csn 58 | Neurosurgery | 0.25 | 5 |
| genotype4 | csn 38 | PCCM | 0.41 | 5 |
| genotype4 | csn 3 | Rehabilitation medicine | 0.19 | 5 |
| genotype4 | csn 48 | ICU | 0.18 | 5 |
| genotype4 | csn 33 | Neurosurgery | 0.46 | 5 |
| genotype4 | csn 23 | Neurosurgery | 0.33 | 5 |
| genotype4 | csn 29 | Neurosurgery | 0.37 | 5 |
| genotype4 | csn 35 | Neurosurgery | 0.47 | 5 |
| genotype4 | csn 64 | ICU | 0.21 | 5 |
| genotype4 | csn 53 | Neurosurgery | 0.22 | 5 |

Supplementary table 2 Data presentation of gene clades, samples, hospital unites, OD value of biofilms and numbers of resistance gene
