# Supplementary material for: Phylogenetic analyses of antimicrobial resistant Corynebacterium striatum strains isolated from a nosocomial outbreak in a tertiary hospital in China
Source: Antonie Van Leeuwenhoek. 2023 Jun 27;116(9):907–18. doi: 10.1007/s10482-023-01855-8 (PMC10371919; doi:10.1007/s10482-023-01855-8)
Supplement: Supplementary file 3 — Supplementary file3 (DOCX 16 kb) [file 10482_2023_1855_MOESM3_ESM.docx]

Supplementary table 3

The difference of hospital unites, OD value of biofilm and numbers of antimicrobial resistance genes among gene clades. (Statistics adopted Fisher's exact test for check before Chi-square test)

| Gene clades | Number（%） | P value |
| --- | --- | --- |
| Clade Ⅰ | 20（31.3） |  |
| Clade Ⅱ | 18（28.1） |  |
| Clade Ⅲ | 11（17.2） |  |
| Clade Ⅳ | 15（23.4） |  |
| Hospital unites |  |  |
| Not Neurosurgery | 34 (53.1) | P＞0.05 (0.126) |
| Neurosurgery | 30 (64.6) |  |
| **OD value (biofilm)** |  |  |
| ＜0.35 | 44 (68.8) | P＞0.05 (0.221) |
| ≥0.35 | 20 (31.3) |  |
| **Numbers of antimicrobial resistance genes** |  |  |
| 1～3 | 4 (6.3) | P＜0.05 |
| 4～6 | 54 (84.45) |  |
| 6～10 | 6 (9.4) |  |
