# Supplementary material for: Phylogenetic analyses of antimicrobial resistant Corynebacterium striatum strains isolated from a nosocomial outbreak in a tertiary hospital in China
Source: Antonie Van Leeuwenhoek. 2023 Jun 27;116(9):907–18. doi: 10.1007/s10482-023-01855-8 (PMC10371919; doi:10.1007/s10482-023-01855-8)
Supplement: Supplementary file 4 — Supplementary file4 (DOCX 16 kb) [file 10482_2023_1855_MOESM4_ESM.docx]

| Supplement table 4 Demographic and clinical features of patients about age, gender, use of antibiotics and underlying diseases in all patients. | |
| --- | --- |
| Data | Numbers (%) |
| **Age(years)** |  |
| ＜50 | 9（13.8） |
| ≥50 | 56（86.2） |
| **Gender** |  |
| Female | 18（27.7） |
| Male | 47（72.3） |
| **Intravenous antibiotics** |  |
| Two or more kinds of antibiotics | 50（76.9） |
| Only an antibiotic | 15（23.1） |
| **Underlying diseases** |  |
| Cerebrovascular disease | 44（67.7） |
| Hypertension | 32（49.2） |
| Pulmonary inflammation | 32（49.2） |
